# Standing in customers’ shoes: How responsible leadership inhibits unethical pro-organizational behavior

**DOI:** 10.3389/fpsyg.2022.1019734

**Published:** 2022-11-29

**Authors:** Ken Cheng, Limin Guo, Yinghui Lin, Panpan Hu, Changchang Hou, Jiaying He

**Affiliations:** ^1^School of Management, Zhejiang University of Technology, Hangzhou, China; ^2^School of Economics and Management, Tongji University, Shanghai, China; ^3^School of Management, Shanghai University, Shanghai, China

**Keywords:** customer-oriented perspective taking, leader competence, responsible leadership, social information processing theory, unethical pro-organizational behavior

## Abstract

Although the negative impact of responsible leadership on employees’ unethical pro-organizational behavior has been documented in the literature, little is known about its underlying processes and boundaries. Drawing on social information processing theory and social learning theory, we built a moderated mediation model to explain why and when unethical pro-organizational behavior could be inhibited by responsible leadership. We conducted a two-phase questionnaire survey to collect data. The empirical results based on the sample of 557 Chinese salespeople showed that customer-oriented perspective taking partially mediated the negative link between responsible leadership and unethical pro-organizational behavior and that leader competence strengthened the direct effects of responsible leadership on customer-oriented perspective taking and unethical pro-organizational behavior as well as the indirect effect of responsible leadership on unethical pro-organizational behavior *via* customer-oriented perspective taking. These findings enrich the current understanding of how responsible leadership relates to unethical pro-organizational behavior, extend the limited literature on customer-oriented perspective taking, and offer some suggestions that managers can follow to inhibit unethical pro-organizational behavior. Limitations and future research directions are also discussed.

## Introduction

Unethical pro-organizational behavior, such as exaggerating the truth about the company’s products to customers and withholding negative information about the company from customers (Umphress et al., 2010), is prevalent in organizations and is harmful to organizational interests (Baker et al., 2019; Mishra et al., 2022). Hence, it is imperative for the organization to find out effective ways to reduce employees’ unethical pro-organizational behavior. Responsible leadership, a leadership style which acknowledges that external stakeholders (e.g., customers) have legitimate claims on organizational activities (Waldman, 2011), has been verified to have an inhibiting effect on employees’ unethical pro-organizational behavior (Cheng et al., 2019; Cheng and Lin, 2020; Inam et al., 2021). Yet, the further exploration of *why* and *when* unethical pro-organizational behavior is inhibited by responsible leadership is rather limited. Law and Jiang (2018) pointed out that unpacking the potential *mediating* and *moderating* mechanisms of a certain relationship could make the causal logic clearer and then contribute to the development of extant theory. We endorse this insightful viewpoint and attempt to narrow the aforementioned gap. Our research questions are twofold: (1) *through what process* can responsible leadership inhibit unethical pro-organizational behavior? (2) *under what condition* can responsible leadership more strongly inhibit unethical pro-organizational behavior?

Extant research has mainly used social learning theory to explain the direct effect of responsible leadership on unethical pro-organizational behavior (Cheng et al., 2019; Inam et al., 2021). Bandura (1977) noted that his social learning theory had drawn much nourishment from the academic thought regarding information processing. In line with previous studies (e.g., Boekhorst, 2015; Usman et al., 2022), we therefore infer that social information processing theory may be very suitable for deeply understanding the potential mechanism through which responsible leadership influences unethical pro-organizational behavior. According to social information processing theory (Salancik and Pfefer, 1978) and relevant research (Wang et al., 2017; Peng et al., 2019; Zhang et al., 2022), environmental cues (e.g., leaders) can influence individuals’ cognitive processes and then shape their behaviors, as these environmental cues can offer individuals necessary guidance for effective functioning within a social context. Given that the mainstream scale of unethical pro-organizational behavior primarily captures customer-oriented unethical pro-organizational behavior (Cheng et al., 2019), we limit our choice of individual cognitive processes to the customer-related ones so as to underscore the unique mechanisms through which unethical pro-organizational behavior is restrained. Customer-oriented perspective taking captures employees’ cognitive process of imagining themselves in a customer’s position and adopting the customer’s viewpoint (Huo et al., 2019). Prior research has suggested that customer-oriented perspective taking can be affected by external factors (e.g., people) and prevent employees from engaging in acts that may damage customers’ interests (Axtell et al., 2007; Ho and Gupta, 2012). Accordingly, we infer that responsible leadership may enhance employees’ customer-oriented perspective taking, which in turn inhibits unethical pro-organizational behavior.

As posited by both social information processing and social learning theories (Bandura, 1977; Salancik, 1977), leaders can exert great effects on employees, but the magnitude of the effects may vary across leaders; in particular, leaders, who have more power, expertise, or other characters that can send admirable and reliable social cues, are more effective in affecting employees. The purpose of responsible leadership is to achieve a good and shared business vision in which the organization builds and cultivates sustainable and trustful relationships to its stakeholders and coordinates them to achieve common goals and business sustainability (Maak and Pless, 2006). This vision is attractive, but its realization may likely be uneasy and will set a great demand on leaders’ capabilities (Muff et al., 2020). Competence is an evaluation that refers to how capable a person is at performing his or her work (Phillips, 1984). Compared to incompetent leaders, competent leaders can obtain employees’ trust more easily and make employees more willing to accept their guidance (Mao et al., 2019). We thus infer that leader competence may function as an important boundary condition of the effects of responsible leadership. That is, competent leaders’ responsible leadership may have greater effects on employees’ customer-oriented perspective taking and unethical pro-organizational behavior.

**FIGURE 1:**
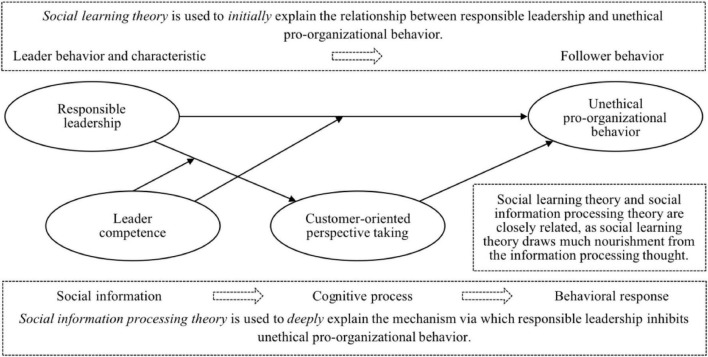
The theoretical model.

We depict the theoretical model in Figure 1 and examine it using a sample of 557 salespeople in China. The results provided support for our theorizing. This study makes a threefold contribution to the literature. First, by verifying the mediating role of customer-oriented perspective taking, we not only provide a clearer account to the question of why responsible leadership could reduce unethical pro-organizational behavior, but also respond to Cheng et al.’s (2019) call for more research on the psychological mechanisms linking responsible leadership to unethical pro-organizational behavior. Second, by proposing and empirically examining the moderating effects of leader competence on the relationships between responsible leadership, customer-oriented perspective taking, and unethical pro-organizational behavior, this study enriches the knowledge about the boundary conditions of the effects of responsible leadership on employees’ cognitive and behavioral reactions. Third, this study extends the literature on customer-oriented perspective taking. On the one hand, we are the first to generate empirical evidence for the positive impact of responsible leadership on customer-oriented perspective taking, thereby shedding light on the important role of leadership in the formation of customer-oriented perspective taking. On the other hand, by demonstrating the negative effect of customer-oriented perspective taking on unethical pro-organizational behavior, we extend the scope of the outcomes of customer-oriented perspective taking in the ethics field (Ho and Gupta, 2012).

## Theory and hypotheses

### Responsible leadership and unethical pro-organizational behavior

Unethical pro-organizational behavior is an inherently paradoxical construct (Lee et al., 2019), which captures unethical behaviors undertaken by employees to potentially benefit the organization and its members (Umphress and Bingham, 2011). Although such behavior is enacted with good intentions, its unethical nature may likely backfire and damage organizational interests (Baker et al., 2019; Mo et al., 2022). Thus, the question of how to restrain unethical pro-organizational behavior has received increased academic attention (Mishra et al., 2022). Specifically, there exist two research streams. One is to explore the inducers of unethical pro-organizational behavior (e.g., organizational identification; Chen et al., 2016), thus appealing to managers to realize the dark side of constructs generally thought to be productive. The other is to identify the inhibitors (e.g., moral identity; Matherne et al., 2018), thus advising managers to strengthen those inhibitors. In the latter research stream, given that leaders usually have great influences on employees, more and more scholars have shifted their attention to the leader-related inhibitors (Cheng et al., 2019; Cheng and Lin, 2020; Inam et al., 2021).

We argue that responsible leadership may inhibit unethical pro-organizational behavior, and we rely on social learning theory to interpret this relationship. A basic tenet of social learning theory is that individuals can learn attitudes and behaviors by observing and imitating others (Bandura, 1977). In the workplace, leaders are an important source of role modeling for employees (Miao et al., 2013). Responsible leadership is a social-relational and ethical phenomenon that occurs in the social process of interaction with stakeholders within and outside the organization (Maak and Pless, 2006). Distinct from other leadership styles (e.g., transformational leadership), responsible leadership broadens the view from a traditional leader–employee relationship to leader–stakeholder relationships and is very concerned about the social targets and objectives of sustainable value creation and positive change (Pless and Maak, 2011; Miska and Mendenhall, 2018). For responsible leaders, making profits for the company and its shareholders is not their only objective. They also care about the interests of other stakeholders (e.g., customers and the society; Voegtlin et al., 2020). For instance, responsible leaders make sure that the products or services are safe and that the real and potential risks are transparently communicated (Maak and Pless, 2006). Hence, under the role model effect of responsible leaders, employees may likely learn what are socially responsible behaviors and reduce socially irresponsible behaviors. Unethical pro-organizational behavior may cause damage to customers (Umphress et al., 2010) and can be seen as a kind of socially irresponsible behavior (Cheng et al., 2019). We therefore infer that responsible leadership may restrain employees’ unethical pro-organizational behavior.

H1: Responsible leadership is negatively related to unethical pro-organizational behavior.

### The mediating role of customer-oriented perspective taking

Customer-oriented perspective taking is a derivative of perspective taking, which reflects a cognitive process of adopting customers’ viewpoints (Axtell et al., 2007; Huo et al., 2019). Extant research has found that customer-oriented perspective taking is a significant factor for the delivery of high-quality service to customers. Specifically, customer-oriented perspective taking has been found to positively affect employees’ proactive service performance (Huo et al., 2019) and behaviors that are beneficial to customers (Axtell et al., 2007; Ho and Gupta, 2012) as well as customers’ satisfaction (Homburg et al., 2009). Social information processing theory suggests that individuals develop their cognitions and attitudes as a function of the social environmental information available to them, which further will guide their behavioral decision (Salancik, 1977; Salancik and Pfefer, 1978). Following this logic and given that leaders are a critical source from which employees gather environmental information (Wang et al., 2017; Peng et al., 2019; Babalola et al., 2020), we infer that responsible leadership of leaders may regulate employees’ cognitive process to make them see things from the perspective of the customer (i.e., customer-oriented perspective taking), which in turn inhibit them from conducting behaviors that are harmful to the customer (e.g., unethical pro-organizational behavior).

Specifically, drawing on social information processing theory and the remarkable work by Ku et al. (2015), we expect that responsible leadership may enhance customer-oriented perspective taking for two reasons. First, employees are more likely to engage in customer-oriented perspective taking when they have an integrated understanding regarding how their work contributes to the customer. If employees clearly know the operating process of their organizations and how their work relates to the bigger picture, they are more able to jump out of the self-focused view and broaden their range of vision (Parker and Axtell, 2001), thus being more likely to see things *via* the customers’ perspective. A very important duty of responsible leadership is to build and cultivate the sustainable relationships with different stakeholders (e.g., customers) and coordinate their actions to realize a good and shared business vision (Maak and Pless, 2006). To perform this duty, leaders need to undertake responsible behaviors toward stakeholders (e.g., trying to achieve a consensus among the affected stakeholders; Voegtlin, 2011). These behaviors are very important environmental cues which may help employees realize that external stakeholders (e.g., customers) are critical to organizational functioning and then prompt employees to stand in customers’ shoes to think about things. Second, employees are more likely to take customers’ perspective when their pro-customer attitudes and motivations are elicited. Responsible leaders emphasize the interests of stakeholders both inside and outside the organization (Maak, 2007). They practice this belief by actively implementing the triple bottom line, integrating multiple perspectives into the decision-making process, and creating desirable values for the related stakeholders (Maak and Pless, 2006). As a kind of social information, these inspiring behaviors of responsible leaders may likely shape employees’ positive attitudes toward customers and motivate employees to care about customers’ interests and take the perspective of the customer.

Further, we expect that customer-oriented perspective taking may inhibit employees’ unethical pro-organizational behavior. Specifically, employees taking the customer’s perspective usually have a clearer understanding of what the customer really needs and expects (Huo et al., 2019) and what negative effects on the customer will occur if they engage in unethical pro-organizational behavior. At the same time, thinking things from the customer’s perspective may likely promote employees’ identification with their customer, as extant research has verified that perspective taking increases perspective takers’ liking and psychological closeness toward the target (Davis et al., 1996). Similar to Ho and Gupta’s (2012) opinion, we deem that employees’ customer identification may drive them to make appropriate decisions to best serve their customer and inhibit them from taking actions that have the potential to cause damage to their customer. One very typical example of these actions is unethical pro-organizational behavior. Prior research on perspective taking found that perspective taking predicted individuals’ deontological decisions (Conway and Gawronski, 2013), thus providing indirect support for our assertion that customer-oriented perspective taking may inhibit unethical pro-organizational behavior. Taken together, guided by social information processing theory and research on perspective taking, we infer that responsible leadership will trigger employees’ customer-oriented perspective taking, which in turn make them less inclined to undertake unethical pro-organizational behavior. That is, customer-oriented perspective taking may function as a mediator in the relationship between responsible leadership and unethical pro-organizational behavior. Yet, considering that there have existed other theoretical perspectives to explain how responsible leadership restrains unethical pro-organizational behavior (e.g., social exchange perspective; Inam et al., 2021), we thus expect customer-oriented perspective taking to partially mediate the aforementioned relationship.

H2: Customer-oriented perspective taking partially mediates the relationship between responsible leadership and unethical pro-organizational behavior.

### The moderating role of leader competence

As a kind of interpersonal perception, competence relates to perceived one’s ability to pursue his or her intentions or bring about desired outcomes (Cuddy et al., 2008). In this vein, leader competence can be understood as the perception regarding how capable a leader is of doing his or her job. Social learning theory posits that role models who have more expertise and admirable characters are more effective in promoting observers’ vicarious learning (Bandura, 1977). Similar viewpoints also appear in social information processing theory and relevant research, which suggest that apart from leaders’ words and deeds, leader competence is also a very important environmental cue in the organizational context that can affect the influences of leaders’ behaviors (Salancik and Pfefer, 1978; Wang et al., 2019). In response to these opinions, numerous studies have verified the moderating effect of leader competence on the influences of leaders’ behaviors on employees (Podsakoff et al., 1983), implying that leader competence is a key boundary of leadership effectiveness. Following this logic, we expect leader competence to moderate the influences of responsible leadership on employees’ customer-oriented perspective taking and unethical pro-organizational behavior.

As a kind of stakeholder-centric leadership, responsible leadership attempts to develop mutual trust between the organization and its stakeholders and ultimately achieve a win-win business vision (Maak and Pless, 2006). These aims are obviously fascinating and inspiring (Cheng et al., 2019). Yet, we should note that the realization of this rosy vision is not going to be a piece of cake, especially in this ever-changing and interconnected business world. In front of employees, responsible leaders not only need to actively participate in stakeholder-related affairs, but also must deal with these affairs well. Otherwise, employees may regard the proposed attractive vision as a daydream and less identify with their leaders. Hence, to ensure the effectiveness of responsible leadership, leader competence is necessary. When employees perceive their leaders as competent, they will trust leaders more (Mayer et al., 1995) and treat leaders as a credible source of guidance (Mao et al., 2019), thus making them more willing to accept their leaders’ influences (Wei et al., 2018). In this case, the stronger leaders’ responsible leadership, the more likely employees will identify with the importance of stakeholders (e.g., customers) and be motivated to care about the interests of stakeholders. Then, employees may engage in more customer-oriented perspective taking and less unethical pro-organizational behavior. In contrast, incompetent supervisors are less effective in influencing employees (Justis et al., 1978). On this occasion, the effect of incompetent leaders’ responsible leadership on employees’ customer-oriented perspective taking and unethical pro-organizational behavior may be limited.

H3: Leader competence moderates the relationship between responsible leadership and unethical pro-organizational behavior such that the relationship is more negative when leaders are perceived as competent than incompetent.

H4: Leader competence moderates the relationship between responsible leadership and customer-oriented perspective taking such that the relationship is more positive when leaders are perceived as competent than incompetent.

The preceding hypotheses propose the mediating effect of customer-oriented perspective taking (i.e., H2) and the moderating effect of leader competence on the responsible leadership–customer-oriented perspective taking relationship (i.e., H4). By combining these two hypotheses, we further propose a moderated mediation hypothesis that the mediating effect of customer-oriented perspective taking will vary based on leader competence and that this mediating effect will be stronger when leaders are perceived as competent than incompetent.

H5: Leader competence moderates the indirect relationship between responsible leadership and unethical pro-organizational behavior through customer-oriented perspective taking such that this indirect relationship is more negative when leaders are perceived as competent than incompetent.

## Methods

### Participants and procedures

Given that some of our key variables (i.e., customer-oriented perspective taking and unethical pro-organizational behavior) are closely related to customers, employees who have the opportunities to contact customers would be very suitable participants in our study. Following prior research (e.g., Ramarajan et al., 2017; Cheng et al., 2019), we chose salespeople as the target sample. It should be noted that in view of the limited financial budget and social resources, it is difficult for us to adopt the random sampling technique to collect data. Therefore, we turned to the convenience sampling technique, a widely used sampling technique in organizational behavior research (e.g., Norton et al., 2017; Guo et al., 2022). Through social relationships, we contacted four companies in Eastern China, involving real estate consulting, insurance, welfare management, and information service industries. The participants were salespeople of these four companies.

To reduce the effect of common method bias, our questionnaire survey employed a two-phase design. At the first phase, with the help of human resource managers, we invited 638 participants to the meeting rooms and randomly distributed them a questionnaire labeled *Part A* and a paired sealed envelope containing a questionnaire labeled *Part B*. The questionnaire *Part A* and the questionnaire *Part B* in pairs had the same matching code which consisted of three capital letters and a three-digit number and was hid in the introduction part of the questionnaire. Then, we informed the participants of the confidentiality and anonymity of our survey and invited them to finish the questionnaire *Part A* that contained items on their direct supervisor’s responsible leadership and leader competence. After the participants put their completed questionnaire into an opaque black box and before they left the meeting rooms, we reminded the participants to take away the paired envelope and keep it safe and sealed until the second-phase survey. To enhance the participants’ emphasis on our surveys, we told them that a bonus worth about two USD would be provide to them if they completed our two surveys. We received 638 questionnaires at the first phase.

Two weeks later, we carried out the second-phase survey. As some salespeople sometimes need to go out to develop business, 64 participants who had attended the first-phase survey failed to join in the second-phase survey. For the rest of the participants, we invited them to the meeting rooms and checked whether their envelope was still sealed. No one had opened the envelope. Then, we invited the 574 participants to open the envelope and finish the questionnaire *Part B* that contained items on their customer-oriented perspective taking, unethical pro-organizational behavior, and demographics. When they completed the questionnaire and put the questionnaire into the opaque black box, they received the bonus. After filtering out the invalid questionnaires (e.g., the ones in which all answers were the same), we obtained 557 usable samples, meaning a final response rate of 87.3%. Among the 557 usable samples, 58.5% were female, and 65.5% had a bachelor’s degree. On average, they were 31.26 years old (*SD* = 3.96) and had 3.01 years of relationship length with their leader (*SD* = 1.46).

### Measures

Unless stated otherwise, all items were measured with five-point Likert scales (1 = *strongly disagree*, 5 = *strongly agree*). The English scales were translated into Chinese following the translation and back-translation procedures suggested by Brislin (1986).

#### Responsible leadership

Responsible leadership was rated with the five-item scale developed by Voegtlin (2011). A sample item is “My direct supervisor considers the consequences of decisions for the affected stakeholders.” To help the participants understand the notion of stakeholder, we explained it in the instruction part of the questionnaire (i.e., “stakeholders are those who may affect or be affected by the organizational actions and policies, such as customers, the public, and so on”). The Cronbach’s α was 0.88.

#### Leader competence

To assess leader competence, we used the six-item scale developed by Mao et al. (2019). A sample item is “My direct supervisor is very capable of performing his/her job.” The Cronbach’s α was 0.87.

#### Customer-oriented perspective taking

Our measure of customer-oriented perspective taking used the four-item scale developed by Axtell et al. (2007). A sample item is “I try to see things from the customers’ viewpoints.” The Cronbach’s α was 0.81.

#### Unethical pro-organizational behavior

Consistent with Tang et al. (2020), we used the four items from the scale developed by Umphress et al. (2010) to measure unethical pro-organizational behavior. A sample item is “I exaggerated the truth about my company’s products or services to customers.” The items were rated from 1 (n*ever*) to 5 (*all the time*). The Cronbach’s α was 0.84.

#### Control variables

Prior research suggested that gender, age, education level, and relationship length with leader have the potential to affect employees’ perspective taking and unethical behaviors (Ramarajan et al., 2017; Cheng et al., 2019). Hence, we controlled for these variables. Gender was measured as one binary variable (1 = male, 2 = female); age and relationship length with the leader were measured in years; education level was divided into three types (1 = technical college or less, 2 = bachelor’s degree, and 3 = master’s degree or above). To account for participants’ tendency to report in a socially desirable way, we followed prior research (e.g., Umphress et al., 2010; Chen et al., 2016) and controlled for impression management by using the 10-item scale developed by Steenkamp et al. (2010). A sample item is “I sometimes tell lies if I have to.” The Cronbach’s α was 0.84.

### Analytic strategy

SPSS 26.0 and Mplus 8.3 were employed to analyze the data. First, we conducted some preliminary analyses. Specifically, we conducted a series of confirmatory factor analyses to test the discriminant validity of our variables as well as the common method bias. Meanwhile, we tested the convergent validity of our variables by calculating their factor loadings, composite reliability (CR), and average variances extracted (AVE). Second, we reported the means, standard deviations, and correlations. To test hypotheses, we used the latent moderated structural equation modeling approach (Sardeshmukh and Vandenberg, 2017) to estimate the path coefficients, simple slopes, indirect effects, and the index of moderated mediation (Hayes, 2015). Meanwhile, we used the Monte Carlo method (Preacher and Selig, 2012) to calculate confidence intervals (CIs) at 95% significance (20,000 repetitions).

## Results

### Preliminary analyses

Table 1 shows the confirmatory factor analysis results. According to Table 1, the four-factor model had better fit (χ^2^ = 198.77, *df* = 146, CFI = 0.99, TLI = 0.99, RMSEA = 0.03, SRMR = 0.03) than other models (i.e., the three-factor, two-factor, and one-factor models), verifying the discriminant validity of our key variables. Moreover, following Podsakoff et al.’s (2003) suggestion, we used the unmeasured latent method factor approach to assess the common method bias. The results showed that adding a common method factor did not result in significant improvements over the model fit indices (χ^2^ = 170.06, *df* = 140, CFI = 0.99, TLI = 0.99, RMSEA = 0.02, SRMR = 0.03), indicating that the common method bias was not a serious issue in our study (Dulac et al., 2008). Furthermore, we tested the convergent validity. As Table 2 shows, all factor loadings were larger than the cutoff rate of 0.60, and the CR for all variables was larger than the threshold value of 0.60 (Hair et al., 2010). At the same time, the AVE for responsible leadership and leader competence was larger than the threshold value of 0.50 (Hair et al., 2010). Although the AVE for customer-oriented perspective taking and unethical pro-organizational behavior was below the benchmark, the factor loadings and CR for these two variables exceeded the threshold values. There also exists some studies that keep variables with AVE below 0.50 (e.g., Safavi and Karatepe, 2018; Zhao and Guo, 2019). Therefore, the convergent validity of our variables can be generally accepted. Besides, the minimum AVE (0.41) was larger than the maximum squared correlation coefficient in all pairs (0.54^2^), which once again confirmed a good discriminant validity of this study (Hair et al., 2010).

**TABLE 1:** The confirmatory factor analysis results.

Model	χ ^2^	*df*	CFI	TLI	RMSEA	SRMR
Four-factor model: RL, LC, CPT, UPB	198.77	146	0.99	0.99	0.03	0.03
Three-factor model: RL + CPT, LC, UPB	551.77	149	0.92	0.90	0.07	0.05
Two-factor model: RL + LC + CPT, UPB	1832.68	151	0.65	0.60	0.14	0.14
One-factor model: RL + LC + CPT + UPB	2217.27	152	0.56	0.51	0.16	0.14

*N* = 557. RL, responsible leadership; LC, leader competence; CPT, customer-oriented perspective taking; UPB, unethical pro-organizational behavior. + represents factors combined.

**TABLE 2:** The convergent validity test results.

Constructs and items	Factor loading	CR	AVE
**Responsible leadership**		0.84	0.51
My direct supervisor demonstrates awareness of the relevant stakeholder claims	0.73		
My direct supervisor considers the consequences of decisions for the affected stakeholders	0.68		
My direct supervisor involves the affected stakeholders in the decision-making process	0.67		
My direct supervisor weighs different stakeholder claims before making a decision	0.75		
My direct supervisor tries to achieve a consensus among the affected stakeholders	0.74		
**Leader competence**		0.87	0.52
My direct supervisor is very capable of performing his/her job	0.73		
My direct supervisor is known to be successful at the things he/she tries to do	0.69		
My direct supervisor has much knowledge about the work that needs done	0.73		
I feel very confident about my direct supervisor’s skills	0.72		
My direct supervisor has specialized capabilities that can increase our performance	0.72		
My direct supervisor is well qualified	0.74		
**Customer-oriented perspective taking**		0.73	0.41
I imagine how things look from the customer’s perspective	0.65		
I think about how I would feel in customers’ situation	0.61		
I try to see things from the customers’ viewpoints	0.66		
I try to imagine myself as a customer in a similar situation	0.63		
**Unethical pro-organizational behavior**		0.78	0.47
I misrepresented the truth to make my organization look good	0.70		
I exaggerated the truth about my company’s products or services to customers	0.64		
I withheld negative information about my company or its products from customers	0.66		
I concealed information from customers that could be damaging to my organization	0.73		

*N* = 557. CR, composite reliability; AVE, average variance extracted.

### Descriptive statistics

Table 3 displays the means, standard deviations, and correlations among variables. In line with our expectations, responsible leadership was negatively related to unethical pro-organizational behavior (*r* = −0.47, *p* < 0.01) but positively related to customer-oriented perspective taking (*r* = 0.54, *p* < 0.01). Customer-oriented perspective taking was negatively related to unethical pro-organizational behavior (*r* = −0.51, *p* < 0.01), thereby providing initial support for our hypotheses.

**TABLE 3:** Means, standard deviations, and correlations.

Variable	*M*	*SD*	1	2	3	4	5	6	7	8
1. RL	3.21	0.94								
2. LC	3.72	0.76	0.20**							
3. CPT	3.24	0.85	0.54**	0.23**						
4. UPB	2.86	0.95	−0.47**	−0.28**	−0.51**					
5. Gender	1.59	0.49	0.09*	–0.06	0.02	–0.04				
6. Age	31.26	3.96	−0.10*	–0.04	–0.03	0.01	–0.03			
7. Education	1.89	0.58	–0.02	–0.01	0.08^†^	–0.02	0.04	0.07^†^		
8. Relationship length	3.01	1.46	0.03	0.01	0.03	–0.06	–0.02	0.48**	0.07^†^	
9. Impression management	3.08	0.63	–0.01	0.17**	–0.05	0.14**	−0.08^†^	–0.07	–0.04	–0.04

*N* = 557. RL, responsible leadership; LC, leader competence; CPT, customer-oriented perspective taking; UPB, unethical pro-organizational behavior. ^†^*p* < 0.10; *p < 0.05; **p < 0.01.

### Hypotheses testing

**FIGURE 2:**
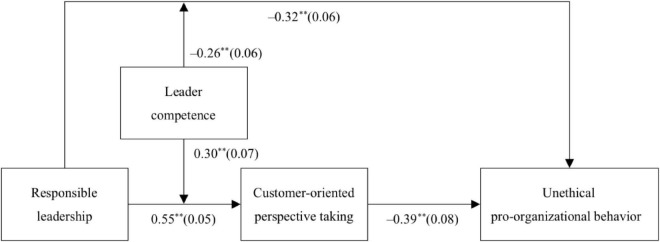
The structural equation modeling path results. *N* = 557. Control variables were included in the model but not shown here for ease of presentation. Values in parentheses were standard error estimates. **p < 0.01.

The latent moderated structural equation modeling approach was used to test our hypotheses. Figure 2 presents the unstandardized path coefficients for the whole model (i.e., the moderated mediation model). According to Figure 2, responsible leadership had a negative and direct effect on unethical pro-organizational behavior (*B* = −0.32, *SE* = 0.06, *p* < 0.01). Meanwhile, responsible leadership was positively related with customer-oriented perspective taking (*B* = 0.55, *SE* = 0.05, *p* < 0.01), which in turn negatively influenced unethical pro-organizational behavior (*B* = −0.39, *SE* = 0.08, *p* < 0.01). Moreover, we calculated the indirect effect of responsible leadership on unethical pro-organizational behavior through customer-oriented perspective taking and estimated its 95% CI by using the Monte Carlo method. The result showed that this indirect effect was significant [*indirect effect* = −0.20, 95% CI = (−0.30, −0.12) which excluded 0]. Thus, H1 and H2 were supported.

**FIGURE 3:**
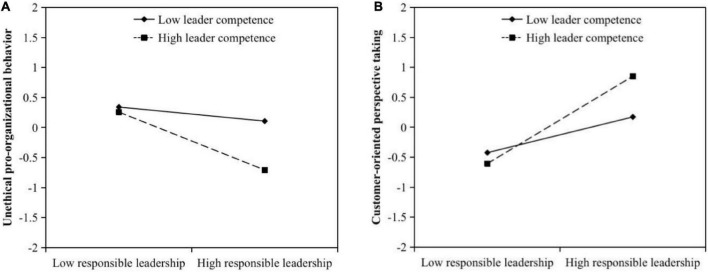
The moderating effects of leader competence on **(A)** the relationship between responsible leadership and unethical pro-organizational behavior and **(B)** the relationship between responsible leadership and customer-oriented perspective taking.

H3 and H4 posited that leader competence would moderate the relationship between responsible leadership and unethical pro-organizational behavior as well as the relationship between responsible leadership and customer-oriented perspective taking. According to Figure 2, the interaction between responsible leadership and leader competence significantly influenced unethical pro-organizational behavior (*B* = −0.26, *SE* = 0.06, *p* < 0.01) and customer-oriented perspective taking (*B* = 0.30, *SE* = 0.07, *p* < 0.01). Then, we plotted these interaction effects by using leader competence at one standard deviation above and below the mean for high and low values, respectively. The interaction effects are depicted in Figure 3. We conducted the simple slope analyses. The results verified the moderating effects of leader competence on the relationship between responsible leadership and unethical pro-organizational behavior (*slope difference* = −0.39, *SE* = 0.10, *p* < 0.01) and the relationship between responsible leadership and customer-oriented perspective taking (*slope difference* = 0.46, *SE* = 0.08, *p* < 0.01): when leaders were perceived as competent, responsible leadership significantly inhibited unethical pro-organizational behavior (*slope* = −0.51, *SE* = 0.10, *p* < 0.01) but promoted customer-oriented perspective taking (*slope* = 0.78, *SE* = 0.07, *p* < 0.01); when leaders were perceived as incompetent, responsible leadership significantly affected customer-oriented perspective taking (*slope* = 0.32, *SE* = 0.05, *p* < 0.01) and only had a marginal effect on unethical pro-organizational behavior (*slope* = −0.12, *SE* = 0.07, *p* < 0.10). Thus, H3 and H4 were supported.

To test H5, we assessed the index of moderated mediation and the indirect effects of responsible leadership on unethical pro-organizational behavior *via* customer-oriented perspective taking at high and low values of leader competence. The 95% CIs of these effects were also estimated by using the Monte Carlo method. The results indicated that leader competence could significantly moderate the aforementioned indirect effect [*index of moderated mediation* = −0.12, 95% CI = (−0.18, −0.06) which excluded 0]. Specifically, the indirect effect was stronger when leaders were perceived as competent [*indirect effect* = −0.30, 95% CI = (−0.43, −0.17) which excluded 0] than incompetent [*indirect effect* = −0.12, 95% CI = (−0.19, −0.06) which excluded 0]. Thus, H5 was supported.

## Discussion

All hypotheses of our research get supported by the relevant analyses. Specifically, we verified that responsible leadership negatively influenced unethical pro-organizational behavior (supporting H1). This finding is not surprising, as prior research has suggested that responsible leaders are often seen as the role models by employees and can show employees what is (un)expected by their behaviors, thus promoting employees’ responsible behaviors and inhibiting employees’ irresponsible behaviors (e.g., unethical pro-organizational behavior; Cheng et al., 2019). In addition, we found that the effect of responsible leadership on unethical pro-organizational behavior is partially mediated by customer-oriented perspective taking (supporting H2). This finding is consistent with the core logic of social information processing theory that environmental cues can shape an individual’s cognitions, which in turn will prompt the individual to undertake corresponding behaviors (Salancik, 1977; Salancik and Pfefer, 1978). Meanwhile, this finding, to some extent, echoes prior research using social information processing theory to explain the mediating role of perspective taking in leaders’ effects on employees (e.g., Wang et al., 2017). Furthermore, we found that leader competence determined the effectiveness of responsible leadership: when leaders were perceived as competent than incompetent, responsible leadership prompted employees to engage in more customer-oriented perspective taking (supporting H3) and (then) undertake less unethical pro-organizational behavior (supporting H4 and H5). This is because a competent leader is usually seen as reliable and trustworthy, and the positive perception of the leader may amplify the influences of leader behaviors on employees’ cognitive and behavioral responses (Podsakoff et al., 1983; Wei et al., 2018; Mao et al., 2019).

### Theoretical implications

The present study contributes to the extant literature in three ways. First, by verifying the mediating effect of customer-oriented perspective taking, we shed new light on the intermediary mechanisms through which responsible leadership inhibits unethical pro-organizational behavior. As noted at the outset, although prior studies have demonstrated the negative influence of responsible leadership on unethical pro-organizational behavior (Cheng et al., 2019; Cheng and Lin, 2020), the psychological mechanisms underlying this association are relatively understudied (Inam et al., 2021). In response to the call for more research on intermediary mechanisms between responsible leadership and unethical pro-organizational behavior (Cheng et al., 2019) and based on social information processing theory (Salancik and Pfefer, 1978), we posited customer-oriented perspective taking as a possible mediator between responsible leadership and unethical pro-organizational behavior. The analytic results based on 557 Chinese salespeople verified our proposition, thereby advancing the current understanding of why responsible leadership restrains employees’ unethical pro-organizational behavior.

Second, the moderating effects of leader competence demonstrated in our study revealed when responsible leadership would have more impacts on employees’ cognitive processes and behaviors. When exploring boundaries of the effects of responsible leadership on employees, prior research has primarily explored the employee-related (e.g., job tenure; Lin et al., 2020) and relationship-related factors (e.g., supervisor-subordinate guanxi; Han et al., 2019). In comparison, leader-related factors have received less attention, although previous research has suggested that leadership effectiveness relies on leader competence to a large extent (Hollander, 1978; Podsakoff et al., 1983). To narrow this research gap, we proposed and examined the moderating effects of leader competence on the relationships between responsible leadership, customer-oriented perspective taking, and unethical pro-organizational behavior, thereby not only enriching the boundaries of the effects of responsible leadership, but also actively responding to the call that when investigating leadership effectiveness, leadership scholars need to pay more attention to its competence boundary (Wei et al., 2018).

Third, the present study adds to the limited literature on customer-oriented perspective taking. Although customer-oriented perspective taking is critical to the provision of high-quality customer service which can attract and retain customers and eventually bring high profits for the organization (Schneider and Bowen, 1995), research on this topic remains limited (Axtell et al., 2007), especially the exploration of how to promote customer-oriented perspective taking (Ramarajan et al., 2017). By verifying the positive impact of responsible leadership on customer-oriented perspective taking, this study extends the antecedents of customer-oriented perspective taking to a very promising research field, namely, leadership. On the other hand, the extant studies on the outcomes of customer-oriented perspective taking have mainly focused on the productive interactions with customers (e.g., helping customers to meet needs; Huo et al., 2019). Less attention has been paid to the counterproductive interactions with customers (Ho and Gupta, 2012), although research in negative event asymmetry has found that negative interactions are more salient than positive interactions in influencing one’s judgments (Taylor, 1991), implying that the counterproductive employee–customer interaction may have a greater effect on customers’ experience. By proving the negative effect of customer-oriented perspective taking on unethical pro-organizational behavior, we narrow this gap to some extent.

### Practical implications

This study brings several implications for management. First, given the negative relationship between responsible leadership and unethical pro-organizational behavior, an effective way for organizations, especially service organizations, to reduce unethical pro-organizational behavior of employees (e.g., salespeople) is to cultivate and strengthen leaders’ responsible leadership. Responsible leadership is a function of the person and the environment (Stahl and Sully de Luque, 2014). Thus, when developing leaders’ responsible leadership, organizations should not only identify leaders’ values and take some measures, if necessary, to modify their values to fit the desired values, but also create and sustain a moral organizational environment to guide the emergence of their socially responsible behaviors.

Second, empirical results showed that customer-oriented perspective taking inhibited unethical pro-organizational behavior. Given this, another way to reduce unethical pro-organizational behavior is to improve employees’ ability to see things from the customer’s perspective. When recruiting and selecting employees, organizations are suggested to assess employees’ customer role orientation, as research has found that employees with high customer role orientation are more inclined to adopt the customer’s perspective (Axtell et al., 2007). After employees have joined in the organization, training and mentoring programs for improving employees’ ability of perspective taking are also advised.

Third, we found that leader competence played a key role in determining the effectiveness of responsible leadership. If leaders are perceived as incompetent, the beneficial effects of responsible leadership will be restrained. To ensure the effectiveness of responsible leadership, how to enhance leader competence is a critical issue that organizations need to address. Muff et al. (2020) suggested that responsible leadership competency contains five dimensions: stakeholder relations, ethics and values, change and innovation, self-awareness, and systems thinking. Organizations can refer to this competency model to develop and train leaders’ competencies of responsible leadership.

### Limitations and future directions

Our study has several limitations. The first limitation is related to the generalizability of our findings, because we solely collected data from the Chinese. Profoundly influenced by Confucianism, Chinese people put great value on one’s virtue and ability. For them, noble characters and excellent abilities are necessary conditions for an authority figure. We thus infer that the magnitude of the interaction of responsible leadership and leader competence on employees may, to some extent, vary across social contexts. We call for future research to examine our model in non-Chinese contexts. The second one concerns the causality. The time-lagged research design that we adopted is unable to make powerful causal inferences, because the data, in its essence, were correlational. Hence, experimental and quasi-experimental research designs are highly recommended in future research. Third, when disentangling the intermediate mechanisms between the relationship between responsible leadership and unethical pro-organizational behavior, we only examined the mediating role of customer-oriented perspective taking *via* the lens of social information processing. Going beyond the cognitive paths, scholars may draw on affective events theory (Weiss and Cropanzano, 1996) and investigate the affective paths *via* which responsible leadership restrains unethical pro-organizational behavior. Fourth, future research may benefit from exploring other moderators beyond leader competence. Enlightened by Liu et al.’s (2012) study, we infer that employees’ attributions to leaders’ responsible leadership may moderate the effects of responsible leadership. When employees attribute leaders’ responsible leadership to the impression management motives, the effects of responsible leadership may likely be weakened.

## Conclusion

In this study, based on social information processing theory and social learning theory, we explored the process through which unethical pro-organizational behavior could be inhibited by responsible leadership, along with an important boundary. Results showed that responsible leadership negatively affected unethical pro-organizational behavior, in part due to customer-oriented perspective taking, and that leader competence strengthened the effects of responsible leadership. These findings enrich the literature on unethical pro-organizational behavior, responsible leadership, and customer-oriented perspective taking as well as provide some practical suggestions that organizations can follow to prevent, control, and reduce employees’ unethical pro-organizational behavior.

## Data availability statement

The data that support the findings of this study are available from KC, chengken@zjut.edu.cn, upon reasonable request.

## Author contributions

KC and LG conducted the conceptualization and data analyses and wrote the first draft of the manuscript. PH conducted the data analyses and critically revised the manuscript for important intellectual content. CH and JH performed the material preparation and data analyses. YL commented on previous versions of the manuscript. All authors substantially contributed to the manuscript and approved the version to be published.
